# Effectiveness and health benefits of a nutritional, chronobiological and physical exercise primary care intervention in fibromyalgia and chronic fatigue syndrome: SYNCHRONIZE + mixed-methods study protocol

**DOI:** 10.1097/MD.0000000000033637

**Published:** 2023-04-28

**Authors:** Noèlia Carrasco-Querol, Gemma González Serra, Nerea Bueno Hernández, Alessandra Queiroga Gonçalves, Marta Pastor Cazalla, Pau Bestratén del Pino, Pilar Montesó Curto, Rosa Caballol Angelats, Immaculada Fusté Anguera, Mª Cinta Sancho Sol, Elisabet Castro Blanco, Anna Vila-Martí, Laura Medina-Perucha, José Fernández-Sáez, M. Rosa Dalmau Llorca, Carina Aguilar Martín

**Affiliations:** a Unitat de Suport a la Recerca Terres de l’Ebre, Fundació Institut Universitari per a la Recerca a l’Atenció Primària de Salut Jordi Gol I Gurina (IDIAPJGol), Tortosa, Spain; b Servei de Rehabilitació i Medicina Física, Hospital de Tortosa Verge de la Cinta i Atenció Primària Terres de l’Ebre, Gerència Territorial de Terres de l’Ebre, Institut Català de la Salut (ICS), Tortosa, Spain; c Institut d’Investigació Sanitària Pere Virgili (IISPV), Tortosa, Spain; d Fundació Institut Universitari per a la Recerca a l’Atenció Primària de Salut Jordi Gol I Gurina (IDIAPJGol), Barcelona, Spain; e Centre d’Atenció Primària Baix Ebre, Direcció d’Atenció Primària Terres de l’Ebre, Institut Català de la Salut (ICS), Tortosa, Spain; f Campus Terres de l’Ebre, Universitat Rovira i Virgili (URV), Tortosa, Spain; g Centre d’Atenció Primària El Temple, Direcció d’Atenció Primària Terres de l’Ebre, Institut Català de la Salut (ICS), Tortosa, Spain; h Unitat d’Expertesa en Sindromes de Sensibilització Central Terres de l’Ebre, Gerència Territorial de Terres de l’Ebre, Institut Català de la Salut (ICS), Tortosa, Spain; i Research group M3O - Methodology, Methods, Models and Outcomes. Facultat de Ciències de la Salut i el Benestar, Universitat de Vic, Universitat Central de Catalunya (UVIC), Vic, Spain; j Universitat Autònoma de Barcelona, Bellaterra (Cerdanyola del Vallès), Spain; k Unitat Docent de Medicina de Familia i Comunitària, Tortosa-Terres de l’Ebre, Institut Català de la Salut (ICS), Tortosa, Spain; l Unitat d’Avaluació i Recerca, Direcció d’Atenció Primària Terres de l’Ebre i Gerència Territorial Terres de l’Ebre, Institut Català de la Salut (ICS), Tortosa, Spain.

**Keywords:** chronic fatigue syndrome, chronobiology, fibromyalgia, multicomponent intervention, nutrition, physical exercise

## Abstract

**Introduction:**

Chronic pain, fatigue and insomnia are classic symptoms of fibromyalgia (FM) and chronic fatigue syndrome (CFS) and seriously affect quality of life. Nutrition and chronobiology are often overlooked in multicomponent approach despite their potential. This study aims to evaluate the effectiveness of a multidisciplinary group intervention based on nutrition, chronobiology, and physical exercise in the improvement of lifestyle and quality of life in FM and CFS.

**Methods:**

Mixed-methods study based on a randomized clinical trial and qualitative analysis with a descriptive phenomenological approach. The study will be conducted in primary care in Catalonia. The control group will follow the usual clinical practice and the intervention group the usual practice plus the studied intervention (12 hours over 4 days). The intervention based on nutrition, chronobiology and physical exercise will be designed considering participants’ opinions as collected in 4 focus groups. To evaluate effectiveness, EuroQol-5D, multidimensional fatigue inventory, VAS pain, Pittsburgh Sleep Quality Index, erMEDAS-17, biological rhythms interview of assessment in neuropsychiatry, REGICOR-Short, FIQR and Hospital Anxiety and Depression Scale questionnaires will be collected at baseline, and at 1, 3, 6, and 12 months post-intervention. Food intake, body composition, resistance and, strength will also be evaluated. The effect size will be calculated using Cohen d and logistic regression models will be used to quantify the impact of the intervention by adjusting for different variables.

**Discussion:**

It expected that the intervention will improve the patients’ quality of life, fatigue, pain and insomnia, as well as food and physical exercise habits, providing effectiveness evidence of a new therapy in addressing these syndromes in Primary Heath Care. Improvements in the quality of life will have a positive socioeconomic impact by reducing health expenditure on recurrent medical consultation, medication, complementary medical tests, etc and favor the maintenance of an active working life and productivity.

## 1. Introduction

Fibromyalgia (FM) and chronic fatigue syndrome (CFS) are considered central sensitization syndromes and involve neurological, metabolic and immunological circuits. Widespread pain, fatigue, and insomnia are the main symptoms of these conditions and seriously affect quality of life, especially in women.^[1]^

The etiopathogenesis of FM remains unclear and controversial,^[2]^ and there are currently no biomarkers or specific medical tests available for it.^[3]^ The prevalence of FM is estimated to be between 0.02% and 6.6% globally^[4]^ and 2.45% in the Spanish population.^[5,6]^ The annual cost per patient in industrialized countries is estimated to be €7256 to €7900, and the indirect costs (loss of productivity) are considered greater than direct ones (medical visits, prescriptions, etc).^[6]^ FM thus represents a high cost for society, estimated at €12.9 million in Spain in 2017.^[7]^

CFS, which often overlaps with FM,^[8,9]^ affects all ages and socioeconomic groups, although it is most common in the 30 to 50 age group. The estimated prevalence in the United States is 0.42%, of which 70% are women.^[10]^ This represents an annual cost of $17 to $24 million.^[11]^ There are no updated data on the incidence of CFS in Europe, but it is estimated to currently affect around 2 million people.^[12]^

The pharmacological treatments currently used in FM are only partially effective, and patients do not improve as expected. The disease becomes chronic and presents in the form of flare-ups, resulting in no positive impact either for patients or the healthcare system. Therefore, recent research on FM treatment focuses on evaluating non-pharmacological multicomponent interventions that involve various disciplines (physical therapy, psychology, and health education).^[6]^ The results of this type of multidisciplinary treatment are positive and have been shown to decrease pain and improve quality of life.^[13,14]^ These interventions were mainly based on cognitive-behavioral therapy, physical activity, and health education, often with very limited or no emphasis on nutrition or chronobiology.

However, there is increasing evidence of the impact of nutrition and chronobiology on different aspects of health and disease, including the impact on central sensitization syndromes such as FM and CFS.^[15–18]^ It is therefore worth including these approaches in the design of non-pharmacological multicomponent interventions. FM has been associated with oxidative stress, mitochondrial dysfunction, and autonomic dysregulation, among other factors. Moreover, several studies associate eating habits, low levels of certain micronutrients (iron, vitamin D, vitamin B12, vitamin B9, vitamin C, vitamin E, essential and branched amino acids, etc), and abnormal body composition to changes in metabolism and energy regulation, and, thus, in the development and impact of FM.^[19–22]^ Other studies show that nutritional interventions aimed at preventing or rebalancing deficiencies of certain micronutrients can improve symptoms, although specific studies and recommendations are lacking.^[1,23,24]^ The relevance of some nutritional deficiencies and low levels of certain nutrients (vitamin C, vitamin B complex, sodium, magnesium, zinc, folic acid, L-carnitine, L-tryptophan, essential fatty acids, coenzyme Q10, etc) has also been highlighted as potentially playing a role in the severity of symptoms associated with CFS.^[25]^

While still scarcely addressed in primary care studies, chronobiology is gaining importance due to the impact it can have in the prevention, triggering and progression of various diseases. Circadian rhythms are particularly relevant in metabolism, hormonal regulation, and cellular repair mechanisms. CFS has recently been associated with various chronobiologic indicators, such as sleep quality, which seem to play a significant role in the development and progression of the syndrome.^[17,18]^ Recent studies also show the impact and relevance of chronobiology in FM.^[26]^ Therefore, chronobiology should be included in depth as an innovative factor in multidisciplinary approaches aimed at preventing and improving chronic diseases. Furthermore, several studies demonstrate the positive impact that approaches involving physical exercise can have on FM and CFS.^[13,17,27]^ Moreover, there are significant interrelationships between nutrition, chronobiology, and physical exercise. The meal times and morning physical exercise contribute to the regulation of circadian clock and, therefore, improve insomnia and fatigue.^[28]^ Exposure to sunlight, especially in the morning, also contributes to the regulation of circadian clock and reduce insomnia.^[29]^ On the other hand, a balanced diet and adequate exercise help to improve body composition and increase muscle mass, which in turn improve metabolism and thus facilitate a more active life with increased exercise and less fatigue.

Although FM and CFS have been associated with certain patterns of food intake and alterations to biological rhythms, most of the studies reviewed to date do not investigate whether these factors play a role in the development of these syndromes or are the result of having them. In any case, an intervention aimed at improving these 2 aspects could have a positive impact on the quality of life of these patients.

This study aims to evaluate the effectiveness of a new short and intensive multicomponent group therapy based on nutritional, chronobiological and physical exercise education. The program will be designed and optimized taking into account the opinions of participants diagnosed with FM, with or without CFS. We designed a complex intervention based on the Medical Research Council methodology, including quantitative and qualitative phases^[30–32]^ so that we can better adapt it to people needs and the setting in which it is implemented. The objective is to encourage patients to adhere to healthy lifestyle habits (nutrition, chronobiology, and physical exercise) to improve fatigue, pain and insomnia and, therefore, quality of life.

## 2. Methods

### 2.1. Study design and intervention description

**Figure 1.**
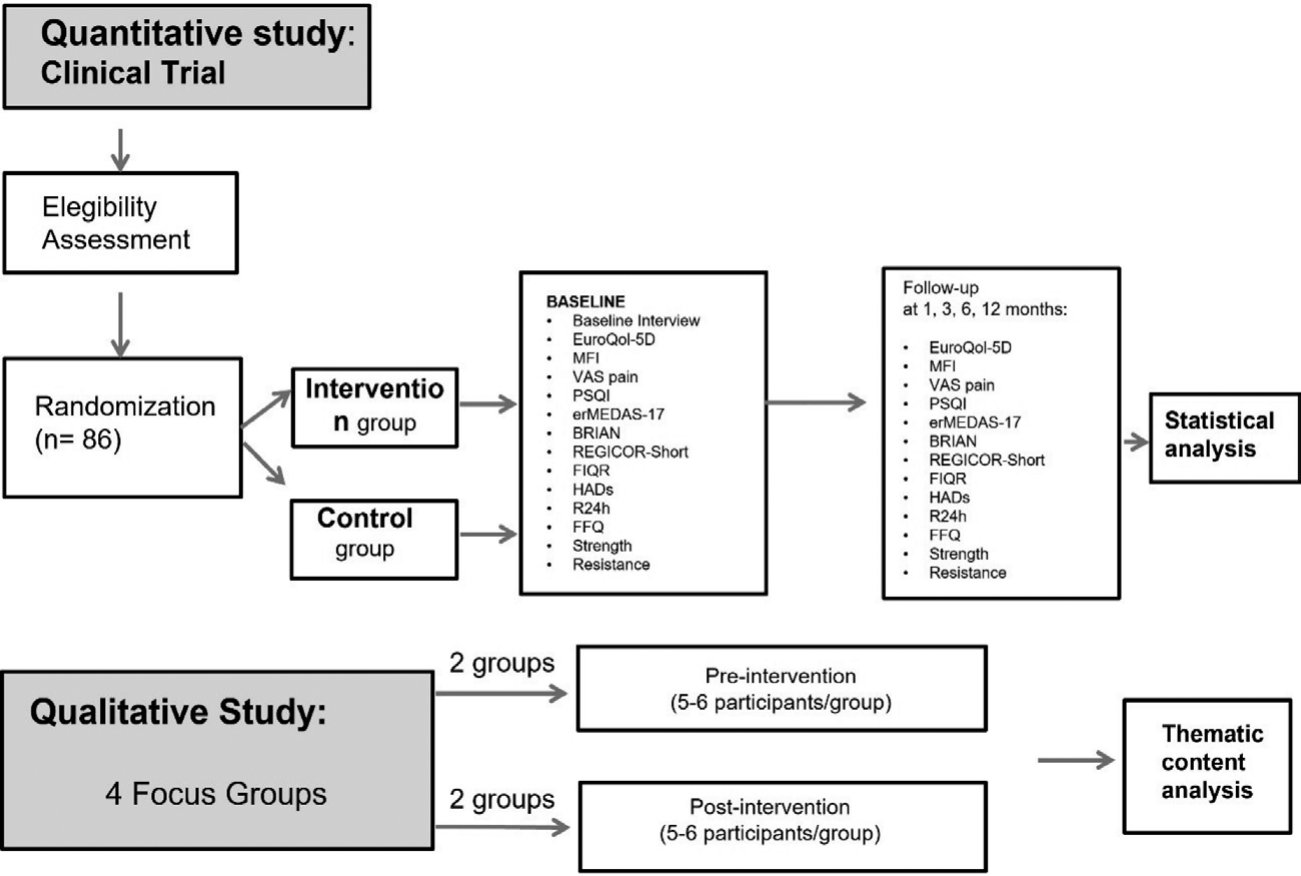
Flow diagram of the study.

Study design: Mixed-methods study that includes a nonblinded, pragmatic randomized clinical trial with parallel groups and a qualitative study with 4 focus groups (two pre-intervention and 2 post-intervention) with a descriptive phenomenological approach. The control group will follow the usual clinical practice, consisting of an individual visit with the FM specialist, who will conduct a standardized medical history and inform the patient of the disease using a custom designed leaflet.^[33]^ The intervention group will follow the usual practice plus the intervention under study (Fig. 1).

**Figure 2.**
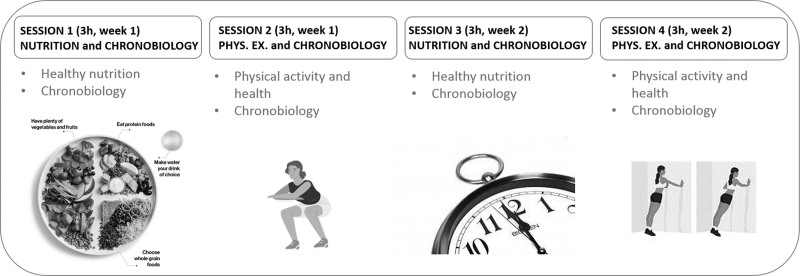
Intervention design: 12 h of healthy habits education (nutrition, chronobiology, and physical exercise) distributed over 2 wk into 3-h sessions 2 d per wk.

Description of the intervention: The intervention to be evaluated is multicomponent and based on actively educating people diagnosed with FM, with or without CFS, on nutrition, chronobiology and physical exercise. It consists of 4 group sessions conducted over 2 consecutive weeks, 6 hours per week on 2 days, 3 hours per day (for a total of 12 hours) (Fig. 2). Data will be collected for both groups and related to quality of life, symptoms, fatigue, pain, nutrition, nighttime rest, body composition parameters, strength, and endurance, before intervention and during follow-up at 1, 3, and 6 months after intervention. The data analysis results will be used to evaluate the effectiveness of the intervention, and the focus group results will be used to optimize the intervention.

### 2.2. Study population

The study subjects of both the quantitative and qualitative phases will be people diagnosed with FM, with or without CFS treated by public primary care centers of the Catalan Health Institute (ICS) in the region Terres de l’Ebre (Catalonia). The International Classification of Diseases-10 was applied^[34]^: M79.7 (FM) and G93.3 (CFS; myalgic encephalomyelitis).

*Study population and inclusion criteria*: People aged 18 to 65 with a recent diagnosis (<10 years) of FM, or a recent diagnosis of FM and CFS, with availability and interest in participating.

*Exclusion criteria*: Not meeting the inclusion criteria, participating in other ICS group interventions aimed at the treatment of these syndromes, presence of severe mental, comorbidity or other relevant medical disorders or pathologies that may interfere with the development of the intervention.

## 3. Quantitative study

### 3.1. Study variables

Main dependent variable: The main variable to be evaluated will be quality of life, assessed with the EuroQol-5D questionnaire.^[35]^

Secondary dependent variables: Fatigue will be assessed with the multidimensional fatigue inventory,^[36]^ consisting of 20 items and several dimensions. Sleep quality and insomnia will be evaluated with the Pittsburgh Sleep Quality Index-19 items.^[37]^ The VAS questionnaire will be used^[38]^ to assess pain. Adherence to the Mediterranean diet will be evaluated with the 17-item erMEDAS questionnaire.^[39]^ Physical exercise and sedentary lifestyle will be assessed with the REGICOR short questionnaire.^[40]^ In addition, circadian biological rhythm will be assessed with the biological rhythms interview of assessment in neuropsychiatry questionnaire,^[41]^ and anxiety with the Hospital Anxiety and Depression Scale.^[42]^ Moreover, people diagnosed with FM will be assessed with the FIQ-R.^[43]^ Food intake will also be evaluated using 24-hour reminders and a FFQ.^[44]^ Body composition will be analyzed through bioimpedance, strength and endurance using various validated tests, such as the 6-min walk test, the 30 second sit to stand test and hand dynamometer measurements.^[45]^

Independent variables: Sociodemographic variables (sex, age, educational level, profession, employment situation, size of city of residence, and comorbidity).

### 3.2. Data collection

Sample size and sampling procedure: The sample size was calculated by GRAMNO V7.12. sample size calculation program and is based on the variations we expect to observe in the EuroQol-5D questionnaire on health-related quality of life.^[35]^ Accepting an alpha risk of 0.05 and a beta risk of <0.05 in a 2-tailed test, a minimum of 43 subjects are needed in each group to detect a minimum difference of 1.6 between 2 groups, with a standard deviation of 1.8. The loss to follow-up rate was estimated at 20%.

Patients diagnosed with FM (ICD-10 code M79.7) with or without CFS (Myalgic encephalomyelitis, code G93.3) treated at Terres de l’Ebre ICS Primary Care (Catalonia) and who meet inclusion criteria will be contacted by phone and invited to participate in the study. Patients will be asked to come to the health center for the first visit, where the study will be explained, and informed consent signatures will be collected. A randomization list will be generated following Efron procedure^[46]^ and using the statistics program SPSS. At this visit, the patient will be informed of which group they will participate in (control or intervention) and the schedule of sessions (or visits). Additionally, the baseline data will be collected from self-administered questionnaires (EuroQol-5D, multidimensional fatigue inventory, VAS pain, Pittsburgh Sleep Quality Index, erMEDAS-17, biological rhythms interview of assessment in neuropsychiatry, REGICOR-Short, FIQR, Hospital Anxiety and Depression Scale) using Microsoft Forms or paper if participants have difficulty using technology. These data will be collected again at 1, 3, 6, and 12 months post-intervention (Table 1). Data will also be collected in person at baseline and at 3 and 6 months post-intervention to evaluate the food intake, body composition, strength and endurance of study participants. These data will be collected from both the intervention and control groups and will be entered into an Excel database for use in the various analyses.

**Table 1:** Data collection planning.

Data collection	Baseline	1m post	3m post	6m post	12m post
To assess quality of life and symptoms, chronobiology and healthy habits (ONLINE):					
*EuroQol-5D: quality of life*	X	X	X	X	X
*MFI (20-items): fatigue*	X	X	X	X	X
*VAS pain: visual analogue pain intensity scale*	X	X	X	X	X
*PSQI (19 items): sleep quality*	X	X	X	X	X
*BRIAN: biological rhythm*	X	X	X	X	X
*REGICOR-Short: practice of physical exercise and sedentarism*	X	X	X	X	X
*Er-MEDAS-17: adherence to Mediterranean diet*	X	X	X	X	X
*FIQ-R: functional impact of fibromyalgia*	X	X	X	X	X
*HADS: anxiety and depression*	X	X	X	X	X
To assess endurance, strength and body composition (in person):					
*Anthropometry* and body composition via bioimpedance and waist circumference	X		X	X	X
Resistance via 6-min walk test	X		X	X	X
Upper trunk strength via dynamometer/handgrip measure	X		X	X	X
Lower trunk strength via 30 s sit to stand test	X		X	X	X
To assess chrononutrition, energy intake and macro- and micronutrient intake (in person):					
24-h reminder	X		X	X	X
FFQ Short	X		X	X	X

BRIAN = biological rhythms interview of assessment in neuropsychiatry, FFQ = food frequency questionnaire, FIQ-R = revised fibromyalgia impact questionnaire, HADS = Hospital Anxiety and Depression Scale, MFI = multidimensional fatigue inventory, PSQI = Pittsburgh Sleep Quality Index.

### 3.3. Statistical analysis

To measure the effect of the intervention within each group, and in the same group before and after intervention, the effect size will be calculated using Cohen d,^[47]^ which classifies effect size as small (0.2–0.5), moderate (0.5–0.8), or large (>0.8). Lastly, logistic regression models will be used to measure the impact of the intervention on improved quality of life by adjusting for any variables that may be considered confounding variables from a clinical point of view.

## 4. Qualitative study

### 4.1. Qualitative data collection

For the qualitative study, data will be collected from 4 focus groups, 2 pre-intervention and 2 post-intervention, comprising 5 to 6 participants with different sociodemographic characteristics to obtain the maximum plurality of speech. Focus groups will be carried out in primary care centers following a thematic guide of questions for intervention participants. The requirements for participation in the pre-intervention focus groups will be to meet the study inclusion criteria. The requirements for participation in the post-intervention focus groups will be to have participated in all the intervention sessions. Convenience sampling will be used and participants will be recruited by phone. In both cases, the participants must agree to participate in the qualitative study and sign the corresponding informed consent. The focus groups will be audio and video recorded, having obtained the required prior consent, and are expected to last no more than 90 minutes. The guide will cover the following themes: general opinion of the effectiveness of the intervention, evaluation of the framework components, aspects to be improved, and the benefits of the intervention on the patient quality of life. Subsequently, the content will be literally and systematically transcribed, always guaranteeing the anonymity of participants, and analyzed using qualitative methods with the aim of improving the intervention.

### 4.2. Qualitative data analyses

A qualitative study with a descriptive phenomenological approach will be conducted. A thematic content analysis will be performed to capture the essential attributes of the participants’ stories. First, literal transcripts will be added to a text corpus, which will include the verbatim transcripts of the focus groups and the field notes of the observers. Emerging themes will be identified based on the grouping of codes (most basic meaningful elements) and categories (clusters of codes). Triangulation of these thematic analyses will be carried out by 2 or 3 members of the research team. An explanatory framework will be developed based on the contributions of the informants. All processes will follow the criteria of rigor in qualitative methodology of Yardley.^[48]^ The intervention design will be adjusted based on the results of the pre-intervention focus groups, and then optimized again based on the results of the post-intervention focus groups, before moving on to the implementation phase.

## 5. Discussion

Primary care is the first line of prevention and early treatment and has great potential in the design, evaluation and implementation of new multidisciplinary interventions for chronic diseases. Central sensitization syndromes, such as FM and CFS, represent a group of chronic pathologies where the pathophysiology is not yet fully elucidated. Therefore, treatment is complex and uncertain. In clinical practice, the number of consultations for these patients does not decrease and there is no perceived improvement despite drug treatment for a significant portion of patients. Therefore, evaluating new non-pharmacological primary care approaches is extremely important. Previous results obtained by the research team pointed to the need to design, evaluate and implement the intervention proposed in this protocol.^[6,49]^ Among the thematic needs identified among participants, nutrition education was one of the relevant topics to be included in new interventions.^[49]^

A new, innovative multicomponent group therapy, based on nutrition and chronobiology, as well as on physical exercise, would mean a significant improvement in the approach to FM and CFS in primary care. In addition, the intervention could have a positive socioeconomic impact: improved quality of life and symptoms for patients, and reduced socioeconomical costs and medical visits, as well as pharmacological prescriptions, as a result of the success of the intervention. As far as we know, this study is new as regards the effectiveness of a multicomponent intervention based on nutrition, chronobiology and physical exercise for patients with FM and CFS in primary care. Furthermore, the intervention design includes participants’ opinions. The proposed intervention could significantly improve clinical care as regards current routine practice.

If the expected benefit of the intervention is confirmed, the results could lead to the updating of primary care clinical guidelines on the treatment of FM and CFS. The results might also help improve public policies related to patient care.

## Acknowledgments

All authors would like to acknowledge the contributions of the ICS, especially the Gerència Territorial ICS Terres de l’Ebre, la Direcció d’Atenció Primària de Terres de l’Ebre, and the Unitat de Sistemes d’ informació de la Gerència Territorial Terres de l’Ebre, who collaborated in the design of this study.

## Author contributions

**Conceptualization:** Noèlia Carrasco-Querol, Gemma González Serra, Alessandra Queiroga Gonçalves, Pau Bestratén del Pino, Rosa Caballol Angelats, José Fernández-Sáez, Carina Aguilar Martín.

**Data curation:** Nerea Bueno Hernández, José Fernández-Sáez.

**Funding acquisition:** Noèlia Carrasco-Querol, Carina Aguilar Martín.

**Investigation:** Noèlia Carrasco-Querol, Gemma González Serra, Marta Pastor Cazalla, Pau Bestratén del Pino, Pilar Montesó Curto, Rosa Caballol Angelats, Immaculada Fusté Anguera, Elisabet Castro Blanco, Anna Vila-Martí, José Fernández-Sáez, M. Rosa Dalmau Llorca, Carina Aguilar Martín.

**Methodology:** Noèlia Carrasco-Querol, Gemma González Serra, Alessandra Queiroga Gonçalves, Marta Pastor Cazalla, Pau Bestratén del Pino, Pilar Montesó Curto, Rosa Caballol Angelats, MªCinta Sancho Sol, Elisabet Castro Blanco, Anna Vila-Martí, Laura Medina-Perucha, José Fernández-Sáez, M. Rosa Dalmau Llorca, Carina Aguilar Martín.

**Project administration:** Noèlia Carrasco-Querol, Nerea Bueno Hernández, Carina Aguilar Martín.

**Resources:** Immaculada Fusté Anguera.

**Supervision:** Noèlia Carrasco-Querol, Alessandra Queiroga Gonçalves, Rosa Caballol Angelats, Immaculada Fusté Anguera, Laura Medina-Perucha, Carina Aguilar Martín.

**Validation:** Noèlia Carrasco-Querol, Carina Aguilar Martín.

**Visualization:** Marta Pastor Cazalla, Pau Bestratén del Pino, MªCinta Sancho Sol, M. Rosa Dalmau Llorca.

**Writing – original draft:** Noèlia Carrasco-Querol.

**Writing – review & editing:** Alessandra Queiroga Gonçalves, Anna Vila-Martí, Laura Medina-Perucha, Carina Aguilar Martín.
